# Erratum: Clinico-Etiological Profile and Predictors of Mortality of Nontraumatic Coma in Children of Upper Egypt: A Prospective Observational Study

**DOI:** 10.4269/ajtmh.20-1540err

**Published:** 2022-12-14

The authors wish to correct errors in “Clinico-Etiological Profile and Predictors of Mortality of Nontraumatic Coma in Children of Upper Egypt: A Prospective Observational Study” (https://doi.org/10.4269/ajtmh.20-1540).

There is a mistake in Table 4 (Clinical features of studied patients at admission in relation to outcome) in the second row (Age 5-10 years), second column (Survivors). The value 27 is correct, but the percentage should be 26.3, not 79.4 as published. The corrected portion of the table appears below.

**Table:** 

Clinical features of NTC (total number = 137)		Survivors, *N* (%), Total = 103	Nonsurvivors, *N* (%), Total = 34	*P* value
Age	< 5 years (61)	41 (39.8)	20 (58.8)	0.038
	5–10 years (29)	27 (26.3)	2 (5.9)	< 0.001
	> 10 years (47)	35 (33.9)	12 (35.2)	0.178

The authors and the *Journal* regret these errors.

